# Physical activity and mental health among Tunisian workers

**DOI:** 10.1192/j.eurpsy.2026.11814

**Published:** 2026-07-31

**Authors:** I. Sellami, A. Abbes, A. Feki, A. Hrairi, M. A. Ghrab, M. L. Masmoudi, M. Hajjaji, K. Jmal Hammami

**Affiliations:** 1 occupational medicine; 2 Rheumatoloy, Hedi Chaker Hospital, sfax, Tunisia

## Abstract

**Introduction:**

Physical activity is a key determinant of mental health, with evidence linking regular activity to reduced stress, anxiety, and depression. However, little is known about this relationship in the Tunisian workforce, where occupational and psychosocial stressors are prevalent.

**Objectives:**

Our study aims to explore the effect of physical activity on mindful self-care, depression, and anxiety among Tunisian workers.

**Methods:**

We conducted a descriptive, analytical, cross-sectional survey among Tunisian workers. We collected sociodemographic, professional characteristics, practice of physical activities, and self-care behaviours using Mindful Self-Care Scale (MSCS). Anxiety and depression were assessed using the Hospital anxiety and depression scale (HADS).

**Results:**

Our population comprised 381 male Tunisian workers with a mean age of 42.4 ± 11.2 years. The mean of tenure of job was 15 ± 10.9 years. According to the HADS, the mean score of anxiety and depression were respectively 6.3 ± 3.6 and 5.4 ± 4. The mean of mindful self-care score was 99.2 ± 34.6. The mean of mindful relaxation, physical care, self-compassion and purpose, supportive relationships, supportive structure and mindful awareness were respectively 10.7 ± 5.8, 18.1 ± 8.2, 16.1 ±8.6, 13.7±7.6, 11.4±6.8 and 11.8±6.7. Physical activity was associated to low score of depression and high score of physical care.

**Conclusions:**

This study highlights that physical activity plays a significant role in improving mental health outcomes and enhancing mindful self-care among workers. Promoting regular exercise can serve as an effective strategy to reduce psychological distress and foster overall well-being.

**Disclosure of Interest:**

None Declared

